# Advanced NGS analysis of cell-free tumor DNA supports clonal relation to primary high-grade B-cell lymphoma lesion and CNS relapse despite MRI negativity

**DOI:** 10.1186/s13000-025-01609-2

**Published:** 2025-02-04

**Authors:** Veronika Navrkalova, Andrea Mareckova, Jakub Porc, Samuel Hricko, Viera Hrabcakova, Jarmila Kissova, Sona Kundova, Marie Jarosova, Sarka Pospisilova, Jana Kotaskova, Andrea Janikova

**Affiliations:** 1 Department of Internal Medicine, Hematology and Oncology, University Hospital Brno and Faculty of Medicine, Masaryk University, Brno, Czech Republic; 2 Center of Molecular Medicine, CEITEC - Central European Institute of Technology, Masaryk University, Brno, Czech Republic; 3 Department of Medical Genetics and Genomics, Faculty of Medicine, Masaryk University and University Hospital Brno, Brno, Czech Republic; 4 Department of Clinical Hematology, University Hospital Brno, Brno, Czech Republic; 5 Clinic of Radiology and Nuclear Medicine, University Hospital Brno, Brno, Czech Republic

**Keywords:** High-grade B-cell lymphoma, Central nervous system involvement, Cell-free DNA, Next-generation sequencing, Integrative diagnostics

## Abstract

High-grade B-cell lymphomas (HGBCLs) are aggressive blood cancers with a severe disease course, especially when the central nervous system (CNS) is involved. Standard histological examination depends on tissue availability and is currently supplemented with molecular tests, as the status of *MYC*,* BCL2*, or *BCL6* gene rearrangements is required for proper lymphoma classification. This case report demonstrates the relevance of cerebrospinal fluid (CSF) cell-free DNA testing by integrative next-generation sequencing (NGS) panel. The benefit of this approach resided in tumor genotyping alongside the proof of CNS progression despite MRI negativity, revealing a clonal relationship with the primary tumor lesion. In addition, our strategy allowed us to classify the tumor as DLBCL/HGBL-*MYC/BCL2* entity. In clinical practice, such a minimally invasive approach provides a more sensitive tool than standard imaging and cell analyzing techniques, enabling more accurate disease monitoring and relapse prediction in particular cases.

**Supplementary Information:**

The online version contains supplementary material available at 10.1186/s13000-025-01609-2.

## Background

High-grade B-cell lymphomas (HGBCLs) represent a group of aggressive non-Hodgkin lymphomas that arise from mature B cells. Typically, patients have dismal clinical outcomes and frequent extranodal involvement, including central nervous system (CNS) impairment [1, 2]. According to the 2022 World Health Organization (WHO) 5th edition of lymphoma classification [3], the term HGBL includes cases with large B-cells different from Burkitt lymphoma (BL) and diffuse large B cell lymphoma, not otherwise specified (DLBCL, NOS), which are characterized by the presence of rearrangements involving the *MYC*, *BCL2* or *BCL6* genes, and 11q aberrations. The proportion of HGBCL among DLBCL tumors is up to 10% [4].

Secondary CNS relapses are not common but clinically challenging complications with devastating consequences. At signs of CNS involvement, brain magnetic resonance imaging (MRI) and lumbar puncture are usually needed. A brain biopsy is inevitable in most cases, although it is often not feasible because of the localization of the tumor. Confirmation of lymphoma invasion by cytology and flow cytometry (FC) examination of cerebrospinal fluid (CSF) is complicated by low numbers of detectable malignant cells [5]. Treatment of such patients is typically based on irradiation of the CNS and systemic chemotherapy using agents able to overcome the blood-brain barrier, but novel approaches indicate benefits for relapsed/refractory patients [1]. However, early detection of CNS relapse in asymptomatic patients is limited. The role of prophylactic chemotherapy in preventing CNS relapse remains a matter of debate [6, 7].

Cell-free DNA (cfDNA) containing circulating tumor DNA (ctDNA), which is found in liquid biopsies (blood or body fluids) of lymphoma patients, is a promising and less invasive approach for genomic tumor profiling at diagnosis or relapse. In the case of CNS involvement, ctDNA analysis from CSF surpasses the search for tumor cells commonly present in limited amounts. cfDNA analysis was proposed as a tool for diagnostics of CNS lymphoma by detecting the L265P *MYD88* mutation, which is known to be present in approximately 80% of CNS lymphomas [8]. Compared with standard methods, cfDNA can be used for disease monitoring, allowing earlier detection of progression [9, 10].

In this case report, we present an advantage of cfDNA analysis using a comprehensive NGS panel, allowing for the simultaneous detection of key genomic features relevant to HGBCL. We aimed to (i) confirm the clonal relationship of the malignant process in CNS to systemic disease, while CT/MRI revealed no lesions in a patient with neurological symptoms, and (ii) assist in the determination of the diagnostic entity. Covering a personalized approach, here we emphasize the need for integrated evaluation of results in specific cases where molecular analysis of liquid biopsy complements standard examinations.

## Case presentation

**Fig. 1:**
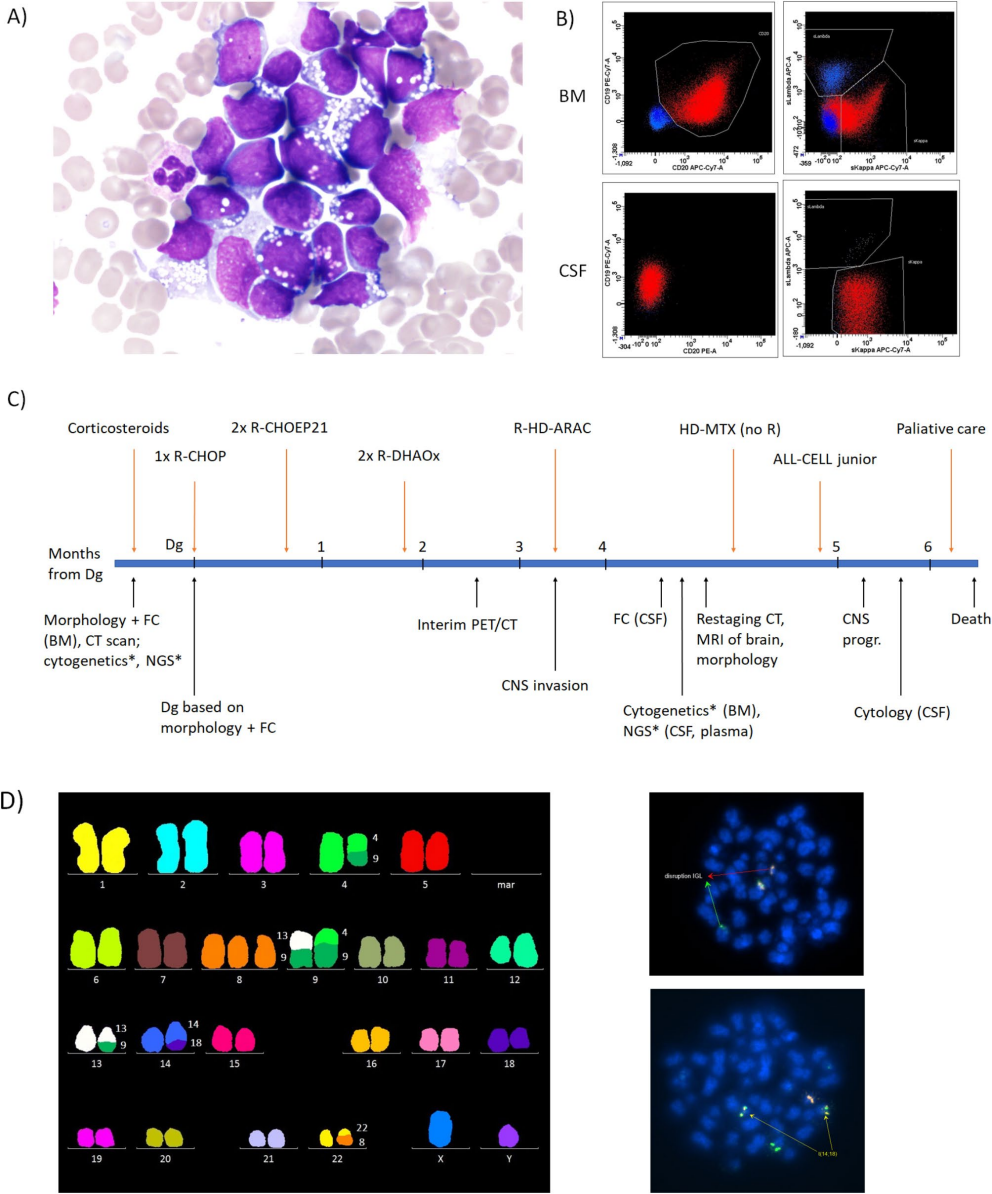
Outputs from standard and supplemental examination of a patient with aggressive lymphoma infiltrating the CNS (except histology that could not be performed): (**A**) morphology of BM infiltrated with lymphoma cells (Burkitt lymphoma-like phenotype); (**B**) flow cytometric analysis showing clonal infiltration of BM and CSF (malignant lymphoma cells in red, non-B cells in blue); (**C**) timeline of various therapy administrations and laboratory examinations (time corresponds with sampling, asterisk marks test performed retrospectively). BM– bone marrow, CNS– central nervous system, CSF– cerebrospinal fluid, CT– computed tomography, FC– flow cytometry, NGS– next-generation sequencing; and (**D**) cytogenetic results of diagnostic BM examination showing complex karyotype by mFISH on the left (47,XY,t(4;9)(q?31.2;p?21),+8,t(8;22)(q24;q11),t(9;13)(p?13;q?14.3),dup(12)(q?q?), der(14)t(14;18)(q32;q21), and IGL and BCL2 rearrangements by FISH on the right (arrows mark chromosomal disruptions).

A 66-year-old man visited the clinic in June 2021 with a sudden onset of physical deconditioning and rapidly progressing exhaustion. He signed the informed consent form for specimen storage, research use, and data publication, which was approved by the University Hospital Brno Ethics Committee. Blood count examination showed thrombocytopenia (10 × 10^9^/l), leukocytosis (30 × 10^9^/l) with 20% blastoid elements, and signs of spontaneous tumor lysis syndrome (lactate dehydrogenase > 120 ukat/l, uric acid 720 µmol/l, creatinine 103 µmol/l, and CRP 69 mg/l). In accordance with the blood population, bone marrow (BM) cytology and flow cytometry, showing 77% of myeloperoxidase-negative blastoid cells with CD5-20+23–38++79b+ immunophenotype in 62% of the cells, suggested aggressive B-cell lymphoma (Fig. 1A and B). Because of rapid clinical deterioration (leukocytosis 70 × 10^9^/l, creatinine 115 µmol/l, lactate dehydrogenase > 150 ukat/l), thrombocytopenia and a low probability of obtaining additional diagnostic information from BM histology, trephine biopsy was not performed. Accurate histological classification (from infiltrated abdominal lymphadenopathy; 80 × 45 mm) distinguishing DLBCL, BL, or HGBCL was not possible due to the dismal progression of the disease. The disease was classified as clinical stage IV according to computed tomography (CT) and blastoid elements present in the BM and peripheral blood (PB).

The patient was pretreated with corticosteroids followed by one cycle of R-CHOP to prevent severe tumor lysis. Intensified chemotherapy included two cycles of R-CHOEP21 (E, etoposide) and two cycles of R-DHAOx (DHAOx, cytosine arabinoside and oxaliplatin) switched due to myelo- and neurotoxicity to one cycle of R-HD-AraC (HD-AraC, high dose cytosine arabinoside) (Fig. 1C). Interim PET/MRI after the first R-DHAOx cycle revealed complete morphologic and metabolic remission (Deauville score of 1).

Two weeks after R-HD-AraC therapy termination, the patient developed diplopia, hypesthesia, tingling, anxiety, insomnia, blurry vision, and left eyelid ptosis. Early progression in the CNS was proven by flow cytometry of CSF, where 176 tumor cells with CD10+CD20- immunophenotype were found in ∼2 ml of CSF. However, a restaging CT scan showed no systemic progression, brain MRI failed to identify any macroscopic lesions, and there was no sign of lymphoma infiltration in the BM. Salvage therapy with HD-MTX (high-dose methotrexate; 3.5 g/m2) was started but without any significant clinical effects. Owing to the leukemia-like clinical behavior of the disease, the patient was switched to an ALL/lymphoblastic lymphoma protocol containing asparaginase (ALL-CELL junior protocol). Regardless of the treatment chosen, the severe neurological symptoms quickly advanced. Rapid progression and chemoresistance led to the planning of palliative whole-brain radiotherapy, but the patient passed away soon (only seven months after diagnosis) at home, outside of the hospital. Thus, an autopsy could not be performed, and information about the death was delayed.

The samples from the time of diagnosis and suspected CNS progression were tested (Fig. 1C). We performed karyotyping, FISH, and mFISH to detect copy number abnormalities (CNAs) and possible translocations in cells obtained from diagnostic BM. Four samples were analyzed by comprehensive next-generation sequencing (NGS) LYNX panel [11] with an updated and validated design (method and target regions are described in the Supplementary data): DNA from a diagnostic BM sample, two cfDNA samples isolated from CSF and plasma from the time of CNS infiltration, and DNA from cells obtained from CSF. The total amount of cfDNA obtained was 14 ng for 10 ml of CSF and 140 ng for 10 ml of plasma. The genomic aberrations and markers detected in various biological materials were then compared.

### Discussion and conclusions

The analysis of diagnostic BM by cytogenetic methods revealed a complex karyotype (CK) with translocation t(14;18)(q32;q21) and variant translocation t(8;22)(q24;q11) (Fig. 1D). The ISCN karyotype identified by karyotyping and confirmed by mFISH was as follows: 47,XY,t(4;9)(q?31.2;p?21),+8,t(8;22)(q24;q11),t(9;13)(p?13;q?14.3),dup(12)(q?q?),der(14)t(14;18)(q32;q21)[20]. CK was present in all evaluated metaphases, and interphase FISH detected *IGH::BCL2* fusion in 59% of the cells (118/200). The follow-up BM sample from CNS relapse showed normal karyotype in 20 evaluated metaphases and negativity for t(14;18) according to FISH.

With the LYNX NGS panel, we detected 16 somatic mutations in the genes *KMT2D*, *BTG1*,* CREBBP*,* BCL2*,* ASXL1*, and *EP300* in the diagnostic BM aspirate. The spectrum of variants is typical for DLBCL, including multiple mutations in one gene [12]. The *ASXL1* gene was an exception, which is affected more frequently in myeloid leukemias [13]. NGS confirmed the presence of the *IGH::BCL2* fusion, CK, and detected two clonal IG light chain rearrangements. The variant t(8;22) was not detected, possibly due to the lower sensitivity of the LYNX panel or the occurrence of breakpoints in nontargeted areas of the *MYC* or *IGL* genes. CNAs included gains on chromosomes 2, 8, and 12; losses on chromosomes 9 and 13; and copy-neutral loss of heterozygosity (CN-LOH) on chromosome 1. The light chain clonotypes involved IGKV2-26 (97%) and IGLV3-22/IGLJ3 (72%) genes. The analysis of cfDNA from CSF in relapse provided results similar to those of diagnostic BM investigation with slightly higher allelic frequencies of mutations and one extra kappa chain rearrangement (IGKV1-5/IGKJ4, 100%). DNA from the cells present in CSF was analyzed separately and showed the same genomic aberrations as cfDNA. cfDNA from the plasma sample gave very different results, with no chromosomal defects or clonality markers. Only the *ASXL1* mutation was detected, suggesting that its origin in clonal hematopoiesis of indeterminate potential (CHIP) is associated with age [14]. It corresponded with cytogenetically normal BM karyotype and clinical observations showing no systemic progression apart from neurological symptoms. Comprehensive NGS testing provided a general view of this patient’s genomic background and markers in various compartments, which are summarized in Table 1.

**Table 1:** Genomic aberrations and their proportions detected by the LYNX NGS panel in various biological materials from a patient with aggressive B-cell lymphoma with CNS involvement during disease. Gene mutation descriptions correspond to MANE (matched annotation from NCBI and EBI) transcripts

Biological material				Bone marrow (DNA)	CSF (cfDNA)	CSF pellet (DNA)	Plasma (cfDNA)
**Gene mutations**	**Gene**	**HGVSc**	**HGVSp**	**Frequency (%)**	**Frequency (%)**	**Frequency (%)**	**Frequency (%)**
	*KMT2D*	c.8936_8937del	p.Leu2979ArgfsTer10	21.7	32.7	29.8	ND
	*KMT2D*	c.7900 C > T	p.Gln2634Ter	49.1	63.3	64.4	ND
	*BTG1*	c.428T > A	p.Val143Glu	34.5	48.5	44.7	ND
	*BTG1*	c.425T > G	p.Met142Arg	34.9	47.2	45	ND
	*BTG1*	c.362T > C	p.Ile121Thr	32.9	47.2	46.3	ND
	*BTG1*	c.90dup	p.Leu31AlafsTer23	34.9	38.2	43.8	ND
	*BTG1*	c.85 A > C	p.Lys29Gln	34.6	34.3	43.4	ND
	*CREBBP*	c.2177_2184del	p.Pro726LeufsTer103	23.2	45.8	39.6	ND
	*BCL2*	c.392 C > T	p.Ala131Val	27.3	36.1	44	ND
	*BCL2*	c.338 C > T	p.Ala113Val	29.5	38.3	44.8	ND
	*BCL2*	c.323 A > G	p.Tyr108Cys	29	47.6	46	ND
	*BCL2*	c.179 C > T	p.Ala60Val	32.4	37.4	51.1	ND
	*BCL2*	c.97G > A	p.Gly33Arg	28.8	34	42.3	ND
	*BCL2*	c.13G > C	p.Gly5Arg	19.5	39.8	40.3	ND
	*ASXL1*	c.1534 C > T	p.Gln512Ter	6.3	3.6	2.3	32.1
	*EP300*	c.3610T > G	p.Cys1204Gly	33	47.3	43.6	ND
**Translocation**	**Gene partners**	**Coordinate**	**Coordinate partner**	**Frequency (%)**	**Frequency (%)**	**Frequency (%)**	**Frequency (%)**
	IGH/*BCL2*	14:105,863,241	18:63,120,802	63	100	96	ND
**Copy number alterations**	**Genome localization (hg38)**	**Type**	**Affected genes (included in LYNX)**	**Frequency (%)**	**Frequency (%)**	**Frequency (%)**	**Frequency (%)**
	chr2:58183800–68,149,300	gain	*XPO1*	70	100	100	ND
	chr8:4617400-145005800	gain	*PAG1*,* UBR5*,* MYC*	70	80	100	ND
	chr12:38525800–73,670,100	gain	*KMT2D*,* STAT6*	80	90	100	ND
	chr1:0-49650114	CN-LOH	*TNFRSF14*,* ID3*	70	100	100	ND
	chr9:21726012–22,249,845	biallelic loss	*CDKN2A*,* CDKN2B*	80	100	100	ND
	chr13:47033917–48,010,829	loss	-	30	80	70	ND
**IG rearrangements**	**Locus**	**V gene**	**J gene**	**Frequency (%)**	**Frequency (%)**	**Frequency (%)**	**Frequency (%)**
inactivating	IGK	IGKV2-26	- (KDE element)	97	91	93	ND
productive	IGK	IGKV1-5	IGKJ4	ND	100	98	ND
unproductive	IGL	IGLV3-22	IGLJ3	72	46	48	ND

cfDNA - cell-free DNA; CN-LOH - copy-neutral loss of heterozygosity; CSF - cerebrospinal fluid; ND - not detected

Although histopathology examination was lacking, the consolidated evaluation of results from molecular cyto/genomic tests suggested that the entity of this particular case was DLBCL/HGBL-*MYC*/*BCL*2 according to the updated WHO classification [3], thus contributing to the proper classification of this lymphoma. Moreover, our results demonstrate that comprehensive NGS analysis combined with less invasive cfDNA sampling allows precise tumor genotyping and the identification of diverse clonality markers, reflecting whole-tumor heterogeneity. It is worth noting that the analysis of liquid biopsies still has some limitations regarding natural cfDNA fragmentation and instability, followed by the need to standardize preanalytical and analytical procedures to reach clinical validity and utility [15, 16].

This report illustrates the advanced use of cfDNA in the diagnostics of aggressive B-cell lymphomas, which enables accurate tumor genetic characterization and supplements standard examinations. Comprehensive NGS analysis of CSF-cfDNA confirmed lymphoma relapse in the CNS in a patient without radiologically detectable lesions and further confirmed the clonal relation to systemic disease. Moreover, increasing knowledge of genomic alterations, clonal heterogeneity, and ctDNA potential should be considered within novel clinical approaches regarding prognosis and potential targeted therapy in aggressive lymphomas. From a future clinical perspective, cfDNA analysis from CSF represents a powerful tool for monitoring CNS involvement and predicting relapse, which is particularly beneficial in cases where other techniques are not sensitive enough to detect malignant cells. Molecular profiling of lymphomas provides a scope for the personalized selection of novel therapeutic strategies.

## Electronic supplementary material

Below is the link to the electronic supplementary material.

Supplementary Material 1

## Data Availability

The original contributions presented in the study are included in the article. The detailed datasets obtained during the study are available from the corresponding author upon reasonable request.

## Abbreviations

ALL: Acute lymphoblastic leukemia
BL: Burkitt lymphoma
BM: Bone marrow
cfDNA: Cell-free DNA
CSF: Cerebrospinal fluid
CHIP: Clonal hematopoiesis of indeterminate potential
CK: Complex karyotype
CNA: Copy number abnormality
CN-LOH: Copy-neutral loss of heterozygosity
CNS: Central nervous system
CT: Computed tomography
ctDNA: Circulating tumor DNA
DLBCL: NOS Diffuse large B-cell lymphoma, not otherwise specified
FC: Flow cytometry
FISH: Fluorescence in situ hybridization
HGBL: High-grade B-cell lymphoma
ISCN: International system for human cytogenomic nomenclature
LYNX: LYmphoid NeXt-Generation Sequencing
mFISH: Multicolor-FISH
MRI: Magnetic resonance imaging
NGS: Next-generation sequencing
PB: Peripheral blood
PET: Positron emission tomography
WHO: World Health Organization
