# Supplementary material for: Advanced NGS analysis of cell-free tumor DNA supports clonal relation to primary high-grade B-cell lymphoma lesion and CNS relapse despite MRI negativity
Source: Diagn Pathol. 2025 Feb 4;20:14. doi: 10.1186/s13000-025-01609-2 (PMC11792325; doi:10.1186/s13000-025-01609-2)

**Supplementary data**

*Cytogenetic examination*

Karyotyping, FISH, and mFISH were performed to detect chromosomal aberrations in bone marrow samples. Twenty cells in metaphase were used for karyotyping. For FISH, 200 interphase cells were evaluated, using XL 22q11 IGL BA probe (MetaSystems GmbH, Germany) for IGL rearrangement and Dual Color Dual Fusion Zytolight Spec BCL2/IGH DCDF probe (ZytoVision GmbH, Germany) to detect *BCL2::IGH* fusion. mFISH analysis was done with MetaSystems GmbH (Germany).

*Integrative NGS analysis by the LYNX panel*

We analyzed four samples of the investigated patient by comprehensive next-generation sequencing (NGS) panel LYNX (Navrkalova *et al.* 2021) with an updated and validated design shown in Figure 1. The panel is capture-based and represents a versatile tool for analyzing various genomic biomarkers in lymphoproliferative disorders – mutations in 67 genes (sensitivity 5%), genome-wide copy number alterations (CNAs and cnLOHs; sensitivity 15-20%), translocations (sensitivity 5%), and antigen receptor rearrangements.

Liquid biopsy samples were collected into special tubes, which preserve cfDNA and prevent genomic DNA release (STRECK, Nebraska, US). Peripheral blood was centrifuged twice (300 g for 20 min, followed by 5000 g for 10 min at room temperature) to obtain plasma samples. CSF sample was centrifuged twice in the same conditions as blood to separate the supernatant for cfDNA extraction and sediment for genomic DNA (gDNA) extraction. CSF and plasma-derived cfDNA were isolated using the QIAamp Circulating Nucleic Acids kit (Qiagen, Hilden, Germany) and controlled on cfDNA ScreenTape assay (Agilent, Santa Clara, CA). gDNA from CSF sediment and bone marrow samples were extracted using the DNeasy Blood and Tissue kit (Qiagen). After quantity assessment (fluorimeter Qubit, Thermo Fisher Scientific Inc., Waltham, MA), NGS libraries were prepared with SureSelect XT HS kit (Agilent Technologies, Santa Clara, CA) according to manufacturer protocol and sequenced in pair-end mode (2x150bp) on the NextSeq instrument (Illumina, San Diego, CA). The genomic aberrations and markers detected in various biological materials were then compared.

*Reference*

Navrkalova V, Plevova K, Hynst J, et al. LYNX (LYmphoid NeXt-generation sequencing) panel: a comprehensive capture-based sequencing tool for the analysis of prognostic and predictive markers in lymphoid malignancies. J Mol Diagn. 2021; Aug;23(8):959-974.

*Figure 1:* Regions of interest and biomarkers included in the updated LYNX panel design.


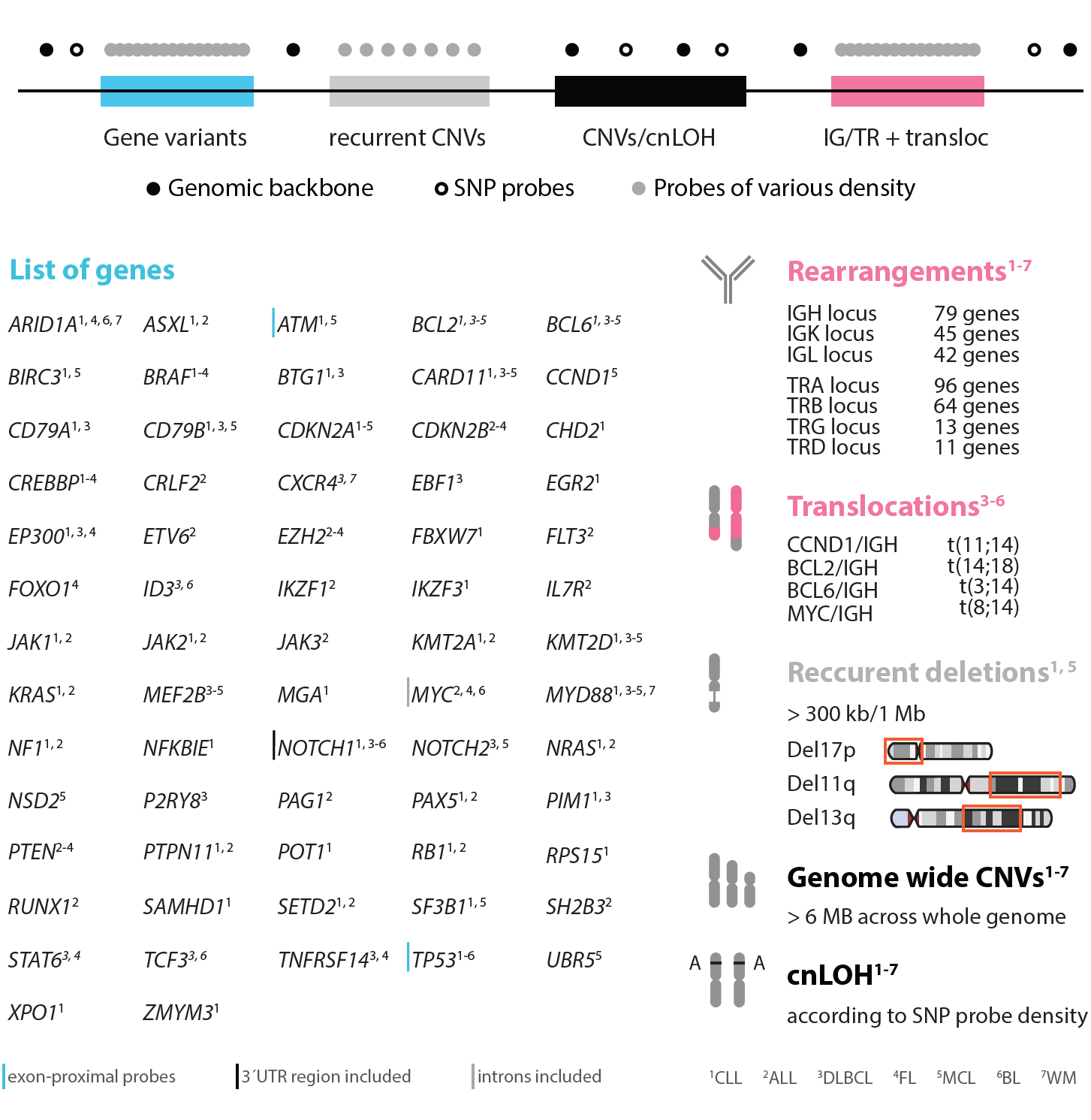

Supplement: Supplementary file 1 — Supplementary Material 1 [file 13000_2025_1609_MOESM1_ESM.docx]
